# C-Src Is Activated by the EGF Receptor in a Pathway that Mediates JNK and ERK Activation by Gonadotropin-Releasing Hormone in COS7 Cells

**DOI:** 10.3390/ijms21228575

**Published:** 2020-11-13

**Authors:** Sarah Kraus, Outhiriaradjou Benard, Zvi Naor, Rony Seger

**Affiliations:** 1 Department of Biological Regulation, The Weizmann Institute of Science, Rehovot 7610001, Israel; kraus_sr@yahoo.com (S.K.); outhiriaradjou.benard@einsteinmed.org (O.B.); 2 Department of Biochemistry, Tel Aviv University, Ramat Aviv 69978, Israel; zvin@tauex.tau.ac.il

**Keywords:** GnRH: GPCR, ERK, JNK, MAPK

## Abstract

The key participants in G-protein-coupled receptor (GPCR) signaling are the mitogen-activated protein kinase (MAPK) signaling cascades. The mechanisms involved in the activation of the above cascades by GPCRs are not fully elucidated. The prototypical GPCR is the receptor for gonadotropin-releasing hormone (GnRHR), which serves as a key regulator of the reproductive system. Here, we expressed GnRHR in COS7 cells and found that GnRHR transmits its signals to MAPKs mainly via Gαi and the EGF receptor, without the involvement of Hb-EGF or PKCs. The main pathway that leads to JNK activation downstream of the EGF receptor involves a sequential activation of c-Src and PI3K. ERK activation by GnRHR is mediated by the EGF receptor, which activates Ras either directly or via c-Src. Beside the main pathway, the dissociated Gβγ and β-arrestin may initiate additional (albeit minor) pathways that lead to MAPK activation in the transfected COS7 cells. The pathways detected are significantly different from those in other GnRHR-bearing cells, indicating that GnRH can utilize various signaling mechanisms for MAPK activation. The unique pathway elucidated here, in which c-Src and PI3K are sequentially activated downstream of the EGF receptor, may serve as a prototype of signaling mechanisms by GnRHR and additional GPCRs in various cell types.

## 1. Introduction

G-protein-coupled receptors (GPCRs) are the largest group of membranal receptors, which transmit signals from a diverse array of external stimuli, including neurotransmitters, hormones, phospholipids, and mitogens. Each of these extracellular agents binds to a specific GPCR, which consequently interacts with a G protein to induce downstream signaling. The G-proteins are heterotrimeric signaling molecules composed of three subunits, namely αβ and γ, which dissociate upon activation to free Gα; and a dimer of Gβγ subunits. Many isoforms of the different subunits have been identified and classified according to the subtype of their α subunit into four groups (Gs, Gi, Gq, and G12). All of these Gα subunits, as well as the dissociated βγ dimer and other receptor-interacting proteins, are capable of initiating diverse downstream signaling pathways [1,2,3,4].

Key components in GPCR-induced intracellular signaling are four groups of mitogen-activated protein kinase (MAPK) cascades, namely extracellular signal-regulated kinase (ERK), c-Jun N-terminal kinase (JNK), p38MAPK, and big MAPK (BMK, also termed ERK5) [5,6,7]. The various MAPK cascades can, under some conditions, be activated by all G proteins. However, each specific G protein seems to use a different mechanism to activate MAPK signaling cascades (reviewed in [8]). Thus, Gαs exerts its downstream effects via adenylyl cyclase/cAMP, which can activate either the cAMP-responsive Ras-guanine nucleotide-exchange factor (Epac [9]) or PKA [10]. Gαq transmits its signals primarily by phospholipase C (PLCβ), IP_3_, and diacylglycerol. The combination of Ca^2+^ and DAG can activate either the protein kinase C (PKC; [11]), which may directly phosphorylate and activate c-Raf [12], or can lead to MAPK activation by a specific Ca^2+^/DAG-GEF [13]. Interestingly, calcium–calmodulin-dependent protein kinase II (CaMKII [14]), as well as several protein tyrosine kinases (PTKs) such as Pyk2, FAK, BTK, and c-Src, have been implicated in Gαq–PLCβ signaling, but their mechanism of action is not yet fully understood [8]. Gα12 operates primarily by stimulation of PTKs [15], Ras-GAP, and PLCε, which directly regulate Ras [16]. Gαi signals can be transmitted via transactivation of receptor tyrosine kinases (RTKs; [17]) or association or activation of Rap-GAP [18]. 

Besides the Gα subunits that seem to be responsible for most of the GPCRs signaling, additional signaling mechanisms towards MAPKs involve the dissociated βγ dimer [19], as well as other signaling molecules. The dissociated βγ dimer may operate via several pathways, including activation of receptor tyrosine kinases [20], direct activation of the protein scaffold protein KSR1 [21], and direct interaction and activation of PI3Kγ. Finally, it has been shown that the receptors may activate signaling proteins, including dynamin [22,23], β-arrestins [4,24], and c-Src [25], which transmit the signals downstream for the activation of MAPK cascades. Importantly, c-Src plays a role in most of GPCRs’ signaling, making it a main signal transducer of most ligands examined.

Gonadotropin-releasing hormone (GnRH) serves as a key regulator of the reproductive system. It acts via a specific GPCR (GnRHR) and triggers the synthesis of the common α and β chains of the gonadotropins, which in turn control the function of the gonads and induce steroidogenesis (reviewed in [8,26,27]). In the pituitary-derived αT3-1 cells, it was shown that GnRHR transmits its signals primarily via Gq, phospholipases, PKCs, and Ca^2+^, culminating in the activation of several MAPK cascades (reviewed in [8,28]). Studies from our laboratories have shown that GnRH in αT3-1 cells involves a direct activation of Raf-1 by PKC, and this step is partially dependent on a second pathway consisting of Ras activation downstream of dynamin and c-Src [29,30,31]. The activation of JNK in these cells is also mediated primarily by PKC, which further induces the sequential activation of c-Src and CDC42/RAC [32]. Interestingly, several additional signaling pathways that can lead from GnRHR to MAPKs were identified in αT3-1 cells as well. Grosse et al. [33] showed that GnRH signals to ERK by activating EGF receptors, while Mulvaney et al. [34,35] showed that ERK is activated in αT3-1 cells via calcium influx through L-type calcium channels and that JNK activation is PKC-independent but mediated by elevated intracellular calcium. On the other hand, Vasilyev et al. [36] showed that in LβT4 cells, short-term incubation with GnRH leads to induction of LHβ transcription, whereas continuous long-term incubation leads to repression of the LHβ transcription. We showed that in LβT2 cells, ERK and JNK are involved in the expression of the LHβ subunit promoter [37]. GnRHR was found to utilize additional distinct intracellular signaling pathways to activate MAPKs. These include PKA [38], independent Gβγ subunits of the Gi/Go proteins [39], and EGF receptor [33,40,41]. Collectively, these results indicate that GnRHR can utilize several signaling pathways in different cell types and under different conditions to execute a single intracellular effect. Therefore, GnRHR serves as a good experimental model to study signaling pathways that can participate in the activation of MAPK cascades by GPCRs.

In the current study, we used GnRHR-expressing COS7 cells and found that both ERK and JNK are activated by GnRH in a similar kinetic manner to that found in αT3-1 cells, but the mechanism that mediates this activation is significantly different. Thus, in the GnRHR-expressing COS7 cells, GnRHR transmits its signals to MAPKs mainly by activating the EGF receptor, although a minor contribution was also detected for the dissociated Gβγ and β-arrestin. JNK activation by GnRHR in these cells is fully dependent on a sequential activation of c-Src and PI3K, which operate mainly downstream of the EGF receptor, but can be activated mildly also by Gβγ. On the other hand, ERK activation by GnRH in these cells is fully mediated by the EGF receptor, which activates Ras directly or via c-Src. This activation is not dependent on the secretion of heparin binding (Hb) EGF, as shown for other GPCRs [42]. Thus, in transfected COS7 cells, GnRH elicits a unique signaling system, in that it places c-Src downstream of the EGF receptor in the pathway from GPCR to MAPK cascades.

## 2. Results

### 2.1. JNK1 Activation by GnRH-a in COS7 Cells

**Figure 1:**
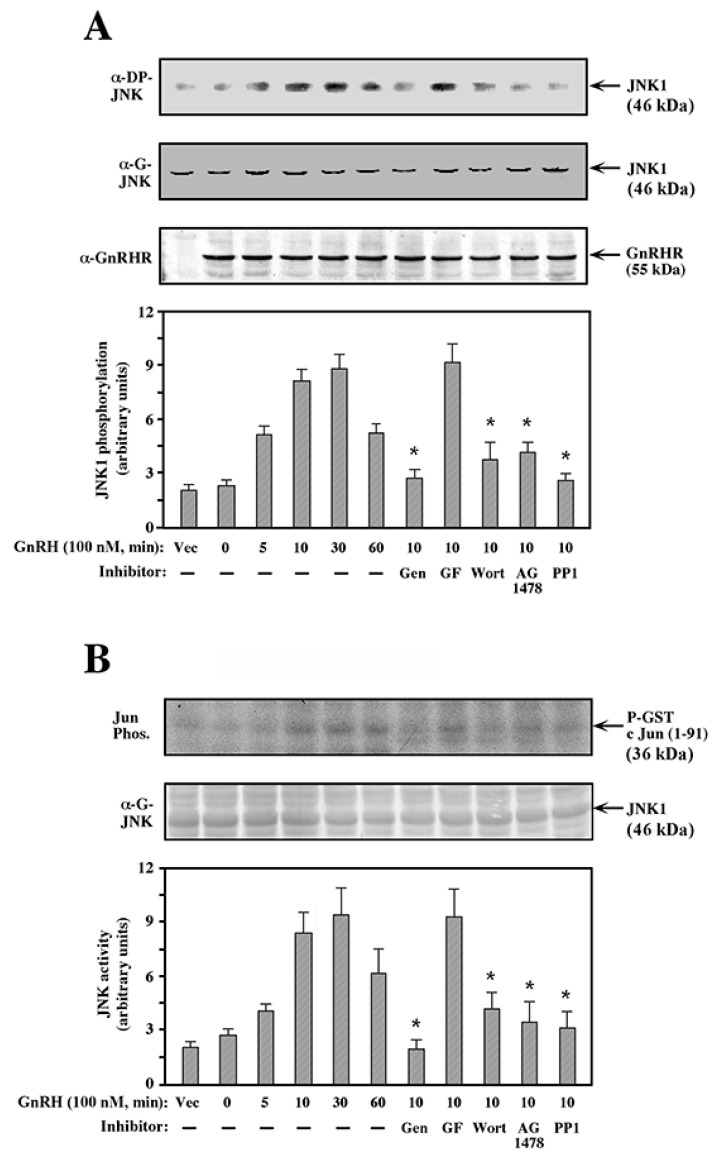
Effects of various inhibitors on JNK1 activation by GnRH-a. (**A**) Phosphorylation of JNK: A plasmid-containing mouse GnRHR was transfected into COS7 cells using the DE-dextran technique as described under Materials and Methods). One plate was transfected with vector alone as control (Vec). This procedure routinely yielded >80% transfected cells as judged by control transfection of plasmid-containing GFP. To ensure similar levels of expression of the GnRHR in all plates, 16 h after transfection, the transfected cells were combined and split into smaller plates (6 cm) for an additional 10 h. The cells were serum-starved (DMEM + 0.1% FCS,18 h) and then were either pretreated (15 min) with 200 μM genistein (Gen), 3 μM GF109203X (GF), 25 nM wortmannin (Wort), 5 μM AG1478, or 5 μM PP1, or left untreated. GnRH-a (10^−7^ M, 10 min) was added to the pretreated and untreated cells (5, 10, 30, and 60 min), or the cells were left untreated as a vector control (Vec. 0). The pJNK1 was determined by Western blot with anti-DP-JNK antibody. Both JNK1 and JNK2 were detected, but the results here are for JNK1 only. The amount of JNK1 was detected with the anti-JNK antibody (α-G-JNK). The results in the bar graph are an average of two experiments. Note: * *p* < 0.05. (**B**) Activation of JNK: The GnRHR-transfected COS7 cells were treated as in (**A**). The activity of JNK towards GST-c-Jun (1–91) was determined (Jun Phos.), as described under Methods. The site of p-GST-c-Jun (1-91) is indicated. The total amount of JNK1 was detected with anti-JNK antibody (α-G-JNK). The bar graphs below are an average of two experiments. Note: * *p* < 0.05.

Signaling by GPCRs is mediated via several distinct pathways, which vary among cell types and stimuli. GnRHR has been proven as a good tool in the study of the GPCR signaling mechanism towards MAPK cascades [8]. To determine the cell-type specificity of GnRH signaling and to study the effect of various signaling inhibitors on this activation, we used COS7 cells that do not express endogenous GnRHR. These cells were transfected with a plasmid-containing mouse GnRHR, which yielded a considerable amount of expression of the GnRHR in most cells, as demonstrated by Western blot analysis with an anti-GnRHR antibody and expression of an unrelated green fluorescent protein (GFP). The efficiency of transfection was more than 80% of the cells in all cases. To ensure a similar level of expression of the GnRHR in all plates of any experiment, the transfected cells were combined and cut into smaller plates. The cells were serum-starved for 16 h prior to the stimulation, pretreated with various pharmacological inhibitors, and then stimulated with 10^−7^ M GnRH-a. The high yield of transfection in COS7 cells allowed detection of endogenous MAPK activation, without a significant background from non-transfected cells. Thus, when examined with anti-doubly-phosphorylated (DP) JNK antibody, which represents JNK activity, a gradual change in recognition by the antibody was observed in two endogenous bands at molecular masses of 46 and 54 kDa, which corresponded to JNK1 and JNK2, respectively. Since the relative amount of staining of the 46 kDa JNK1 was stronger than that of the 54 kDa JNK2, we demonstrate here only the results of JNK1. Thus, expression of GnRHR in COS7 cells without an addition of GnRH-a did not change the activity of JNK1. However, elevation in JNK1 phosphorylation was already detected 5 min after stimulation with GnRH-a, which peaked at 10–30 min after stimulation and declined thereafter (Figure 1A). Pretreatment of the cells with the PTK inhibitor, genistein, as well as inhibitors of PI3K (wortmannin), EGF receptor (AG1478), and c-Src (PP1) but not the PKC inhibitor, GF109203X, significantly reduced the activation of JNK1 by GnRH-a. Similar results to those obtained with the anti-DP-JNK antibody were observed when the endogenous JNK activity was measured by an in vitro kinase assay (Figure 1B). Again, genistein, wortmannin, AG1478, and PP1 significantly prevented JNK activation by GnRH-a, while GF109203X had no inhibitory effect. This pattern of inhibition is markedly different from that obtained in αT3-1 cells, where stimulation of JNK activity with GnRH-a was inhibited by GF109203X but not by wortmannin or AG1478 [32], indicating that the JNK activation by GnRH may differ in different cell lines.

### 2.2. Involvement of the EGF Receptor, βγ Dimer, c-Src, and β-Arrestin in JNK1 Phosphorylation by GnRH-a

**Figure 2:**
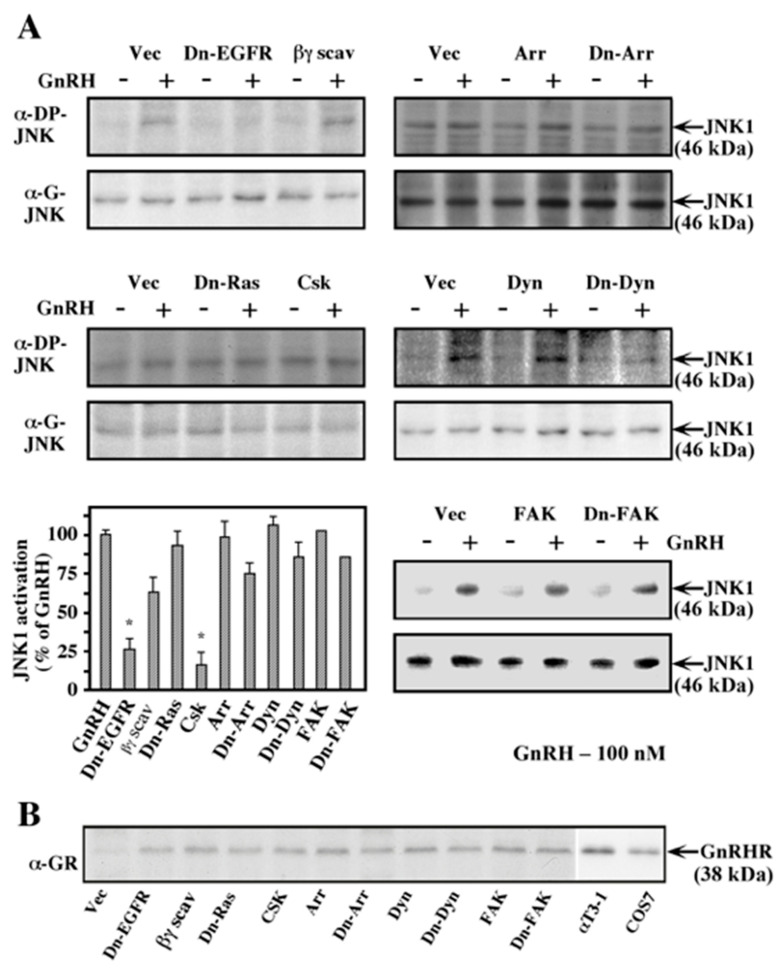
Effect of various signaling components on JNK1 activation by GnRH. (**A**) Involvement of signaling components. COS7 cell were co-transfected with plasmid-containing mouse GnRHR, together with each of the following plasmids: K721A-EGF receptor (Dn-EGFR); CD8-tagged βARK (βγ scav); N-17 Ras (Dn-Ras); Csk-pRK5 (Csk); β-arrestin2 (Arr); V54D-β-arrestin2 (Dn-Arr); dynamin (Dyn); K44A-dynamin (Dn-Dyn); human FAK (FAK); and N-terminally truncated FAK (Dn-FAK). Two days after transfection, the cells were serum-starved for 16 h and then either treated with GnRH-a (10^−7^ M; 10 min, +) or left untreated (-). Activated JNK1 was determined with anti-DP-JNK antibody (α-DP-JNK). The amount of total JNK1 was detected with anti-JNK antibody (α-G-JNK). The results in the bar graphs represent percent activation of that obtained in the GnRH-a stimulated cells that were co-transfected with GnRHR and vector control in each experiment. The results are average of three experiments. Note: * *p* < 0.05. (**B**) GnRHR expression in transfected COS7 cells. The amount of transfected GnRH was detected by an anti-GnRHR antibody. The two right lanes are from a different blot.

In order to study the possible involvement of additional signaling components in the GnRHR–JNK pathway and to confirm the involvement of components that were identified by the inhibitors above, we co-expressed GnRHR together with interfering mutants of various signaling components into COS7 cells. Serum starvation and treatment with GnRH-a were followed as described above. As expected from the inhibition with AG1478 and PP1, the dominant negative form of the EGF receptor, as well as Csk, which inhibits the activity of c-Src, nearly abolished the activation of JNK1 by GnRH-a (Figure 2A). β-Arrestin, which can serve as a mediator of signaling of GPCRs towards MAPKs [24,43], seemed to play a minor role in the GnRHR-JNK signaling. Although the wild type β-arrestin had no significant effect on JNK1 activation by GnRH-a, the dominant negative form of this protein inhibited this activation by ~30%. Similar inhibition was exerted by CD8-tagged βARK, which acts as a scavenger for the dissociated βγ dimer [44]. On the other hand, dominant-negative Ras, as well as the wild type and the dominant negative forms of FAK and dynamin, did not seem to influence the studied pathway. Taken together, these results suggest major roles for the EGF receptor, c-Src, and PI3K and minor roles for β-arrestin and βγ dimer in the pathway that links the GnRHR to JNK in the transfected COS7 cells. Other known signaling components such as PKC, FAK, dynamin, and Ras do not seem to be involved in this process. As seen in Figure 2B, the amount of transfected GnRHR was roughly similar in all experiments and to the amount of the receptor in αT3-1 cells. This similarity was consistent in all of the experiments, indicating that the number of receptors in the transfected COS7 cells was not too high, which make the results more reliable.

### 2.3. ERK Activation by GnRH-a in COS7 Cells

**Figure 3:**
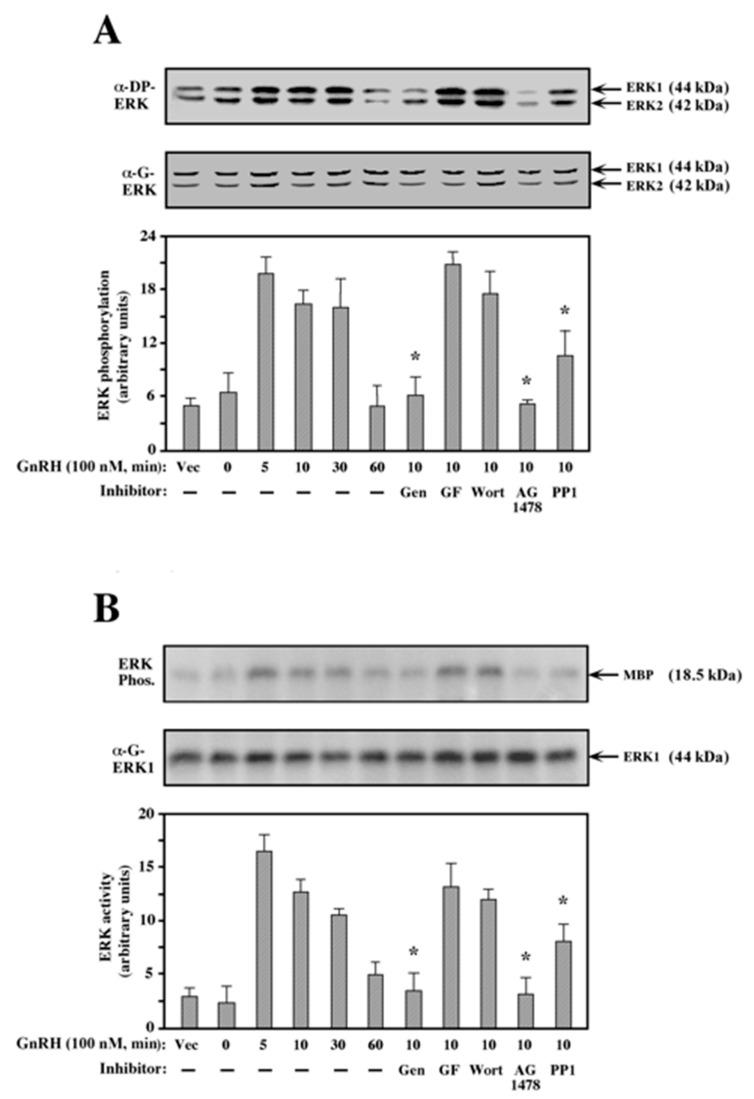
Effect of various inhibitors on ERK activation by GnRH-a. (**A**) Phosphorylation of ERK: A plasmid-containing mouse GnRHR was transfected into COS7 cells using a DE-dextran technique (Materials and Methods), and the cells were treated as described in the legend to Figure 1. After serum starvation, the cells were either pretreated (15 min) with 200 μM genistein (Gen), 3 μM GF109203X (GF), 25 nM wortmannin (Wort), 5 μM AG1478, or 5 μM PP1, or left untreated. GnRH-a (10^−7^ M, 10 min) was added to the pretreated and untreated cells (5, 10, 30, and 60 min), or the cells were left untreated as a vector control (Vec. 0). Phosphorylation of ERK1 and ERK2 was detected with anti-DP-ERK antibody (α-DP-ERK). The total amounts of ERK1 and ERK2 were detected with the anti-ERK antibody (α-G-ERK). The results in the bar graphs below are an average of three experiments. Note: * *p* < 0.05. (**B**) Activation of ERK: The GnRHR-transfected COS7 cells were treated as in (A). The activity of ERK towards myelin basic protein (MBP) was determined (ERK Phos.) as described under the Methods section. The total amount of ERK was detected with the anti-ERK antibody (α-G-ERK); the site of ERK1 migration is indicated. The bar graphs below are an average of three experiments. Note: * *p* < 0.05.

We then studied the mechanism of ERK activation by GnRH in the transfected COS7 cells. As above, the cells were serum-starved and treated, and anti-DP-ERK antibody was used to detect the phosphorylation of ERK in its activation loop. As with JNK1, the expression of GnRHR in COS7 cells did not change the level of the regulatory phosphorylation of endogenous ERK1 and ERK2 (Figure 3A). The addition of GnRH-a to the transfected cells resulted in a substantial phosphorylation of both ERK1 and ERK2, which peaked at 5 min after treatment, remained high for an additional 25 min, then declined 30 min later. Pretreatment of the cells with the PTK inhibitor genistein, AG1478, and to some extent also with PP1, inhibited the GnRH-a-induced phosphorylation of ERK. On the other hand, treatment with the PI3K inhibitor wortmannin and the PKC inhibitor GF109203X had no inhibitory effect. Similar results were also observed when the endogenous ERK activity towards MBP was measured by an in vitro kinase assay (Figure 3B). Again, ERK was transiently activated by GnRH-a, with a peak at 5–10 min after stimulation. Genistein and AG1478 completely prevented the GnRH-a-induced ERK activation, PP1 inhibited this activation by ~35%, while GF109203X and wortmannin had no detectable inhibitory effect. As with JNK1, this pattern of inhibition is different from that obtained in αT3-1 cells, where stimulation of ERK by GnRH-a was inhibited by GF109203X but not by AG1478 [30]. Thus, the pathways that lead to ERK activation by GnRH-a differ in the different cell lines. Moreover, the sensitivity of ERK activation to inhibitors in the transfected COS7 cells was different from the sensitivity of JNK to the same inhibitors. This indicates that the pathway that leads to ERK activation by GnRH is different, at least in part, from the one leading to the JNK cascade.

**Figure 4:**
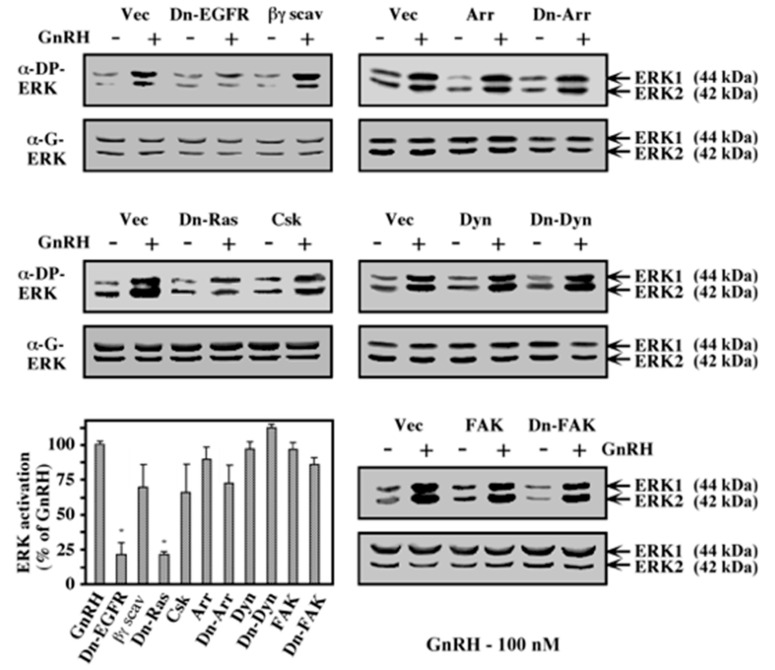
Effect of various signaling components on ERK activation by GnRH. COS7 cells were co-transfected with plasmid-containing mouse GnRHR, together with each of the following plasmids: K721A-EGF receptor (Dn-EGFR); CD8-tagged βARK (βγ scav); N-17 Ras (Dn-Ras); Csk-pRK5 (Csk); β-arrestin2 (Arr); V54D-β-arrestin2 (Dn-Arr); dynamin (Dyn); K44A-dynamin (Dn-Dyn); human FAK (FAK); and N-terminally truncated FAK (Dn-FAK). Two days after transfection, the cells were serum-starved for 16 h and then either treated with GnRH-a (10^−7^ M; +) or left untreated (-). Phosphorylated ERK 1 and ERK2 were detected with anti-DP-ERK antibody (α-DP-ERK). The amounts of total ERK1 and ERK2 were detected with anti-ERK antibody (α-G-ERK). The results in the bar graph represent the percent activation of that obtained in the GnRH-a-stimulated cells that were co-transfected with GnRHR and vector control in each experiment. The results are averages of three experiments. Note: * *p* < 0.05.

To study the possible involvement of additional signaling components in the GnRHR to ERK pathway, we co-expressed the GnRHR together with interfering mutants of various signaling components in COS7 cells. As expected from the study using pharmacological inhibitors, the dominant negative form of EGF receptor significantly attenuated the phosphorylation of ERK1 and ERK2 upon GnRH-a treatment (Figure 4). In addition, the expression of dominant-negative Ras, which acts upstream of the ERK cascade in many systems [45], also caused a substantial reduction in GnRH-a-stimulated ERK phosphorylation. Csk, which inhibits c-Src activity, reduced the GnRH-stimulated phosphorylation of ERK by ~40%. As observed for JNK1, the dominant negative form of β-arrestin inhibited this activation by ~30%. On the other hand, the Gβγ scavenger CD8–βARK, as well as the wild type and the dominant negative forms of dynamin and FAK, had no effect. Taken together, the results suggest a role for the EGF receptor, Ras, c-Src, and possibly also β-arrestin in the pathway that links the GnRHR to ERK in transfected COS7 cells. Other known signaling components such as PKC, FAK, dynamin, PI3K, and dissociated βγ dimer do not seem to be involved in this process.

### 2.4. Ras Activation by GnRH-a is Mediated by the EGF Receptor and c-Src

**Figure 5:**
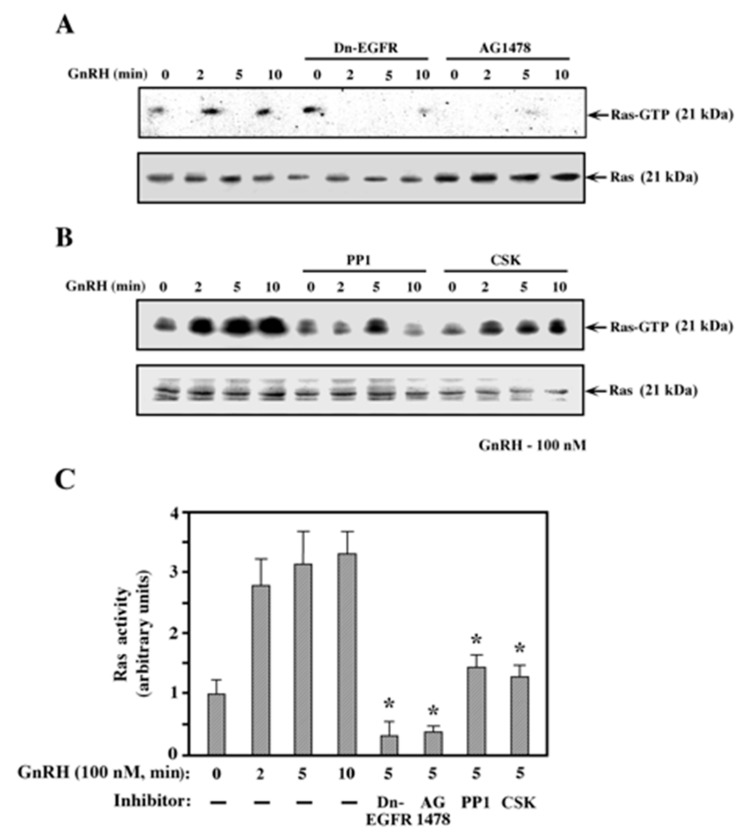
Mechanism of Ras activation by GnRH. COS7 cells were co-transfected using the DE-dextran method with 5 μg of the GnRHR together with 5 μg Dn-EGFR (**A**), Csk (**B**), or vector control. Thirty-two hours after transfection, the cells were serum-starved for 16 h, those that were co-transfected with vector controls were either left untreated or pretreated with AG1478 (5 μM, **A**) or PP1 (5 μM, **B**) for 15 min, then all of the plates were treated with GnRH-a for various time periods. Activated Ras-GTP was determined by Ras precipitation using GST-RBD as described under the Materials and Methods section. These results were reproduced three times. (**C**) Quantification of the results in (**A**) and (**B**). The results in the bar graph represent fold activation of that obtained in untreated cells. These are averages of three experiments. Note: * *p* < 0.01.

One of the most important mediators of signals to the ERK cascade is the small GTP binding protein Ras. Therefore, we used the Raf-RBD pull down assay [46] in order to detect the Ras activation by GnRH. As expected, Ras was activated within 2 min after GnRH-a addition to the transfected COS7 cells, the activity peaked at 5 min, then decreased thereafter. Interestingly, when the cells were co-transfected with dominant-negative EGF receptor or preincubated with the EGF receptor inhibitor AG4178, both basal activity and GnRH-a-stimulated activity were almost completely abolished (Figure 5A). On the other hand, co-transfection with Csk or preincubation with the Src inhibitor PP1 also caused reductions (50% ± 10%, Figure 5B) in Ras activation, but their effect was significantly lower than that of the inhibitors of the EGF receptor. Therefore, it is likely that the EGF receptor is the main stimulator of Ras, while c-Src may mediate part of the EGF-receptor-induced signal and the well-established Grb–Sos pathway probably mediates the other part of the signal [8].

### 2.5. Role of EGF Receptor in the Mediation of GnRH Signals

Our results show that EGF receptor plays a central role in the transmission of GnRHR-initiated signals towards JNK and ERK. Another signaling component that is involved in this process is c-Src, which seems to be a central player in the pathway that leads to JNK activation and participates to some extent also in the GnRHR-ERK pathway. It became important to study the activation of these two components and the interplay between them.

**Figure 6:**
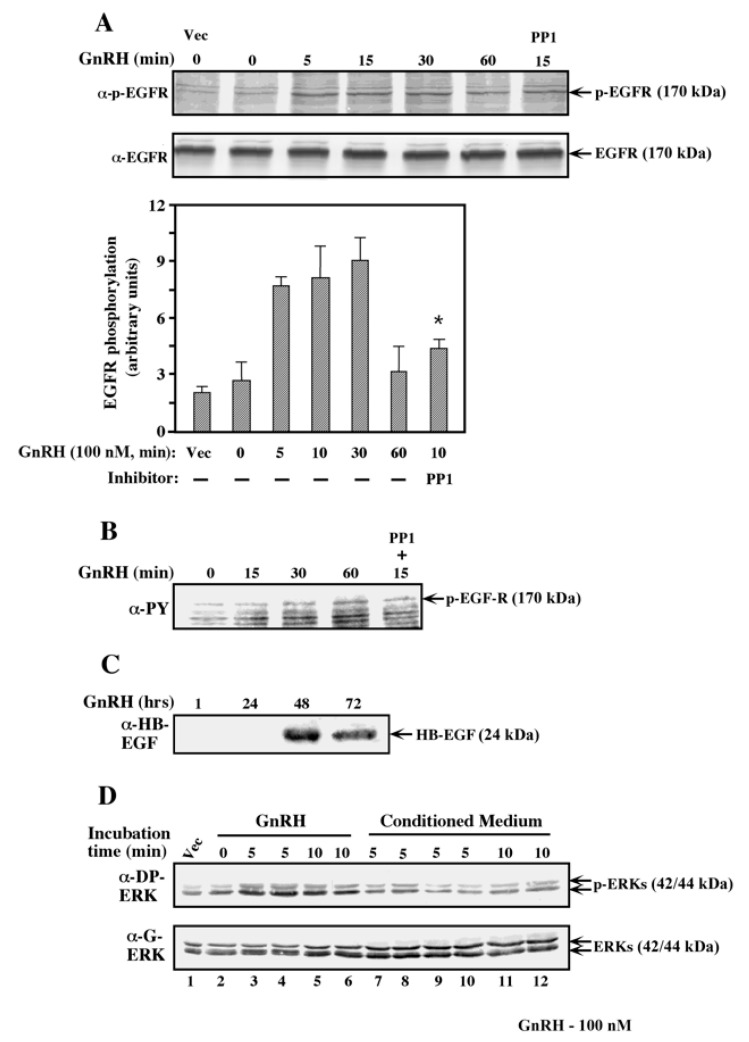
EGFR transactivation by GnRH. (**A**,**B**) COS7 cells that were transiently transfected and serum-starved as described were stimulated with GnRH-a (10^−7^M) for the indicated times and tested for enhanced tyrosine phosphorylation using anti-phospho EGF receptor (**A**). The results were quantitated and the bar graph represents averages of three experiments. Note: * *p* < 0.05 PP1 inhibition compared to 15 min without inhibitor. (**B**) The results were confirmed with anti-pY antibody. (**C**) Detection of Hb-EGF shedding in COS7 cells expressing GnRHR. Conditioned medium (CM) was collected and incubated with heparin-agarose to precipitate the Hb-EGF ectodomain. Hb-EGF secreted in the culture medium was detected by Western blot analysis using goat anti-Hb-EGF antibody. (**D**) ERK activation in non-GnRHR-expressing COS7 cells treated with CM from GnRH-treated GnRHR-expressing cells: COS7 cells were transfected as described, serum-starved for 16 h, and treated with GnRH-a (10^−7^M) for 5 and 10 min. CM from transfected cells (lanes 1–6) was collected and transferred to non-transfected COS7 cells (lanes 7–12) that did not express the GnRHR, then further incubated for the indicated times. Non-transfected cells stimulated (5 min) with CM from untreated transfected cells or cells transfected with Vector alone were used as controls (lanes 7, 8). Phospho-ERK was determined with anti-DP and anti-general-ERK antibodies.

Using both anti-pEGF receptor and anti-PY antibodies, we showed that EGF receptor was rapidly activated by GnRH (Figure 6A,B), and remained active for more than 60 min. This activation was not affected by inhibitors of c-Src, indicating that this PTK is not located upstream of the EGF receptor, as suggested in several other systems [47,48]. A main mechanism for GPCR-induced activation of the EGF receptor seems to act through activation of a membranal proteinase (e.g., MMP9), which in turn releases the membrane-bound Hb-EGF to activate the receptor [49]. We examined the amount of Hb-EGF released to the medium upon activation of the GnRHR-transfected COS7 cells and found that it could not be detected in the first day after GnRH-a addition but appeared at later time points after stimulation (Figure 6C). In order to verify the lack of Hb-EGF, we used the conditioned medium of GnRH-activated COS7 cells to activate the MAPK in non-transfected COS-7 cells, as described in [42]. Indeed, the conditioned medium did not induce any activation of ERK (Figure 6D), indicating that the activation of the EGF receptor is not mediated by Hb-EGF.

### 2.6. Activation of c-Src by GnRH-a is Mediated Mainly by the EGF Receptor

**Figure 7:**
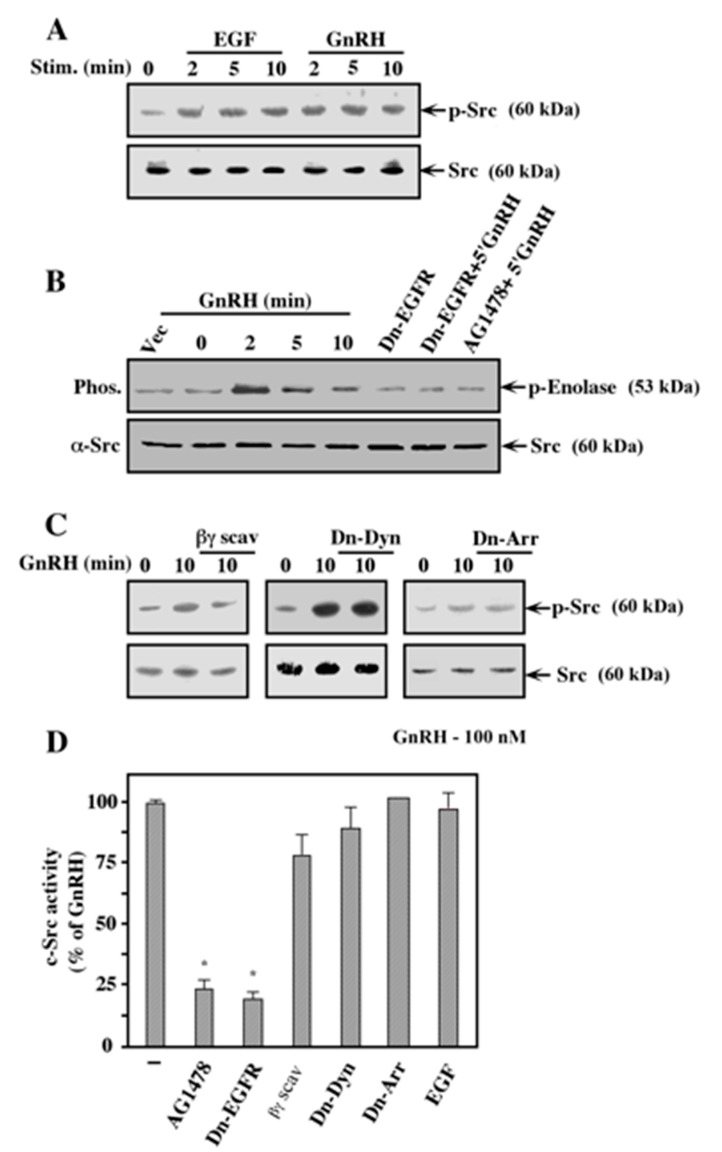
Mechanism of c-Src activation by GnRH. (**A**) COS7 cells were transfected with a plasmid-containing mouse GnRHR. Thirty-two hours after transfection, the cells were serum-starved for 16 h and one plate was pretreated with PP1 (5 μM, 15 min). Then, the cells were stimulated either with EGF (50 ng/mL) or GnRH-a (10^−7^ M) for the indicated times. Phosphorylation of c-Src on Tyr 416 (activated c-Src) was detected with anti-phospho-Src antibody (p-Src). The amount of c-Src did not change throughout the experiment, as detected by anti-c-Src antibody (Src). These results were reproduced twice. (**B**) COS7 cells were co-transfected with mouse GnRHR, together with either K721A- EGF receptor (Dn-EGFR) or with a vector control. One plate was transfected with vector alone (Vec). Thirty-two hours after transfection, the cells were serum-starved for 16 h, after which one plate was pretreated with AG1478 (5 Μm, 15 min). Then, the cells were stimulated with GnRH-a (10^−7^ M for the indicated times) and c-Src activity towards denatured enolase was determined as described under the Methods section. The amount of immunoprecipitated c-Src was determined using anti-c-Src antibody (lower panel). The results were reproduced 3 times. (**C**) COS7 cell were co-transfected with plasmid-containing mouse GnRHR together with plasmids containing either CD8-tagged βARK (βγ scav), K44A-dynamin (Dn-Dyn), V54D-β-arrestin2 (Dn-Arr), or no insertion as control. Two days after transfection, the cells were serum-starved for 16 h and then either treated with GnRH-a (10^−7^ M; 10 min) or left untreated (0). Activation of c-Src was determined by Western blot analysis using anti-phospho-c-Src antibody (p-Src) or anti-general-Src antibody (Src). These results were reproduced twice. (**D**) The amount of activated c-Src was determined by densitometry and plotted as a bar graph of the percentage activation against that of GnRH-a-stimulated cells that were co-transfected with GnRHR and vector control in each experiment. These results represent averages and standard errors of 3 experiments. Note: * *p* < 0.05.

The results above clearly indicate that c-Src does not act upstream of the EGF receptor. Therefore, we aimed to elucidate the role of the PTK in mediating the GnRH signals. Interestingly, we found that treatment of the GnRHR-expressing COS7 cells resulted in a sustained activation of c-Src, similarly to its activation upon GnRH-a treatment (Figure 7A). In both stimulations, activation of the PTK was seen within 2 min of treatment. The addition of AG1478 or co-transfection with the dominant negative form of the EGF receptor substantially reduced c-Src activation of GnRH-a (Figure 7B). A reduction in GnRH-a-induced c-Src activation was also seen with the Gβγ scavenger, however the dominant negative forms of dynamin and β-arrestin had no effect (Figure 7C,D). No effect was detected with the PKC inhibitor GF109203X. These results suggest that the mode of c-Src activation in this system mainly involves activation downstream of the EGF receptor, which is likely complemented by a signal via the dissociated Gβγ. On the other hand, PKC, which is the key player in c-Src activation by GnRH in αT3-1 cells [32], does not participate in this activation at all in this system. Similarly, dynamin and β-arrestin do not seem to participate in the activation of c-Src either, indicating that the mechanism involved in the activation is unique to the GnRHR-expressing COS7 cells.

### 2.7. Activation of PI3K/PKB by GnRH-a Occurs Downstream of c-Src

**Figure 8:**
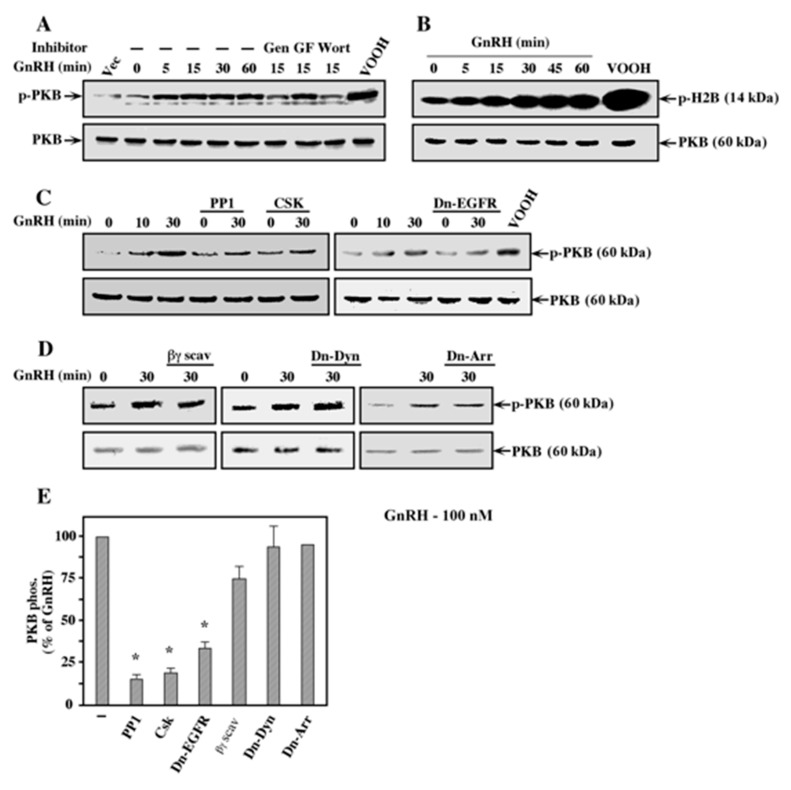
Mechanism of activation of PI3K/PKB by GnRH-a. (**A**) COS7 cells were transfected with a plasmid-containing mouse GnRHR. Thirty-two hours after transfection, the cells were serum-starved for 16 h, after which the plates were pretreated with either 200 μM genistein (Gen), 3 μM GF109203X (GF), or 25 nM wortmannin (Wort). Then, the cells were either stimulated with GnRH-a (10^−7^ M for the indicated times) or with the non-specific activator peroxovanadate (200 µM H_2_O_2_, 100 µM vanadate; VOOH). Cellular extracts of these cells were subjected to a Western blot analysis using either anti-phospho-PKB antibody (Ser 473; p-PKB) or anti-PKB antibody (PKB). These results were reproduced twice. (**B**) COS7 cell were treated as in (**A**) and then subjected to immunoprecipitation with anti-PKB antibody followed by an in vitro phosphorylation reaction with histone H2B as a substrate (p-H2B). The phosphorylation was detected by autoradiography and the amount of immunoprecipitated PKB was detected with anti-PKB antibody (PKB). These results were reproduced twice. (**C**) COS7 cell were co-transfected with plasmid-containing mouse GnRHR together with plasmid-containing Csk, K721A-EGF receptor (Dn-EGFR), or no insertion as vector control. Thirty-two hours after transfection, the cells were serum-starved for 16 h, after which two plates were pretreated with the c-Src inhibitor PP1 (5 μM, 15 min). Then, the cells were stimulated with GnRH-a (10^−7^ M for the indicated times) and phosphorylation of PKB was detected with either anti-phospho PKB (p-PKB) or with anti-PKB (PKB) antibodies. These results were reproduced 3 times. (**D**) COS7 cells were co-transfected with mouse GnRHR, together with either CD8-tagged βARK (βγ scav), K44A-dynamin (Dn-Dyn), or V54D-β-arrestin2 (Dn-Arr), or no insertion as vector control. Two days after transfection, the cells were serum-starved for 16 h and then either treated with GnRH-a (10^−7^ M; 30 min) or left untreated (0). Activation of PKB was determined by Western blot analysis as in (C). The results were reproduced twice. (**E**) Activation of PKB was determined by densitometry. The bar graphs represent percentage activation from GnRH-a stimulated COS7 cells that were co-transfected with GnRHR and vector control in each experiment. The results represent averages and standard errors of 3 experiments. Note: * *p* < 0.05.

As shown above, PI3K is involved in the pathway that lead to JNK activation of GnRH-a, but does not participate in the GnRH-a–ERK pathway in the GnRHR-transfected COS7 cells. Previous studies have shown that PI3K can transmit signals of GPCR, where it is activated mainly by the dissociated Gβγ dimer [50], or downstream of PTKs [8]. Since PKB is activated downstream of PI3K, we used the phosphorylation of this protein kinase as a readout for PI3K activation. To detect the activity of PKB, we used both an in vitro kinase assay with histone H2B as a substrate and anti-phospho PKB antibody, which demonstrate a good correlation with the activity of the kinase. Our results show that the phosphorylation and activity of PKB were already seen 5 min after GnRH-a treatment, and both were gradually increased up to 55 min later (Figure 8A,B). We then used several inhibitors of putative upstream components and found that genistein and wortmannin, but not GF109203X, abolished the phosphorylation of PKB (Figure 8A). Next, we examined whether crosstalk between the EGF receptor, c-Src, and PI3K may play a role in transmitting GnRH-a signals. Indeed, we found that pretreatment of the cells with the c-Src inhibitor PP1, as well as co-transfection of the inhibiting Csk, resulted in a substantial decrease in PKB activation by GnRH-a (Figure 8C). Therefore, it is likely that the GnRHR–PKB pathway is mostly regulated by c-Src in our system.

We then co-transfected the cells with dominant-negative EGF receptor, which significantly reduced the activation of PI3K/PKB by GnRH as well (Figure 8C), but this reduction was only partial (~70%). Importantly, a partial inhibitory effect on PKB activation was caused by the Gβγ dimer (Figure 8D), while the dominant negative forms of dynamin and β-arrestin had no influence on this stimulation process (Figure 8D). Taken together, our results suggest that JNK activation by GnRH is mediated by a pathway that includes a sequential activation of c-Src, PI3K, and JNK. In addition, the upstream machinery involved in c-Src stimulation by GnRH in the GnRHR-transfected COS7 cells is mediated mainly by EGF receptor, a process that is likely complemented by the dissociated Gβγ dimer.

### 2.8. Involvement of Gαi in the GnRH-a-Mediated Signaling

**Figure 9:**
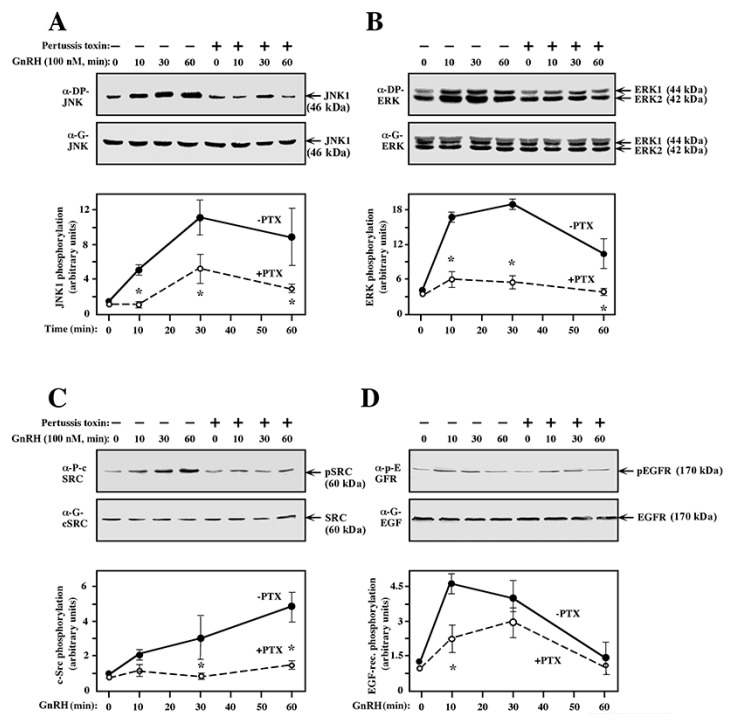
The effects of the pertussis toxin on the phosphorylation of ERK, JNK, c-Src, and EGF receptor via GnRH. COS7 cell were transfected with a plasmid-containing mouse GnRHR. Thirty-two hours after transfection, the cells were serum-starved for 16 h. The cells were then either pretreated with pertussis toxin (100 ng/mL, 5 h, open circles, dashed lines) or left untreated (filled circles, solid lines). GnRH-a (10^−7^ M, indicated times) was added to the pretreated and untreated cells, or the cells were left untreated as a control (time 0). Phosphorylation of JNK1 (**A**), ERK (**B**), c-Src (**C**), and EGF receptor (**D**) was determined using the appropriate anti-phospho and anti-general antibodies. The results in the graphs below are average and standard errors of three experiments. Note: * *p* < 0.05.

Although our results indicate that EGF receptor is the main upstream mediator of GnRH signaling, they do not supply information on the mechanism by which this receptor is activated upon GnRH treatment. Previous studies on αT3-1 cells showed that GnRHR may transmit its signal via Gαq and PLCβ [8]. However, this is clearly not the case for the GnRHR-transfected COS7 cells, as no PKC involvement was detected in the GnRH-a-induced activation of MAPKs, indicating that another G protein plays a role in this system. To test this point, we used pertussis toxin, which is a known selective inhibitor of Gαi [51]. Thus, GnRHR-transfected COS7 cells were serum starved followed by pretreatment with the toxin for 5 h. Then, the cells were treated with GnRH-a and the phosphorylation of JNK, ERK, c-Src, and EGF receptor was examined by Western blots with the proper antibodies. Interestingly, GnRH-a induced elevated phosphorylation of all these signaling proteins, which was significantly reduced by the pertussis toxin (Figure 9). On the other hand, the pertussis toxin did not affect either the expression of MAPKs, c-Src, and EGF receptor, or the viability of the GnRHR transfected COS7 cells. Thus, these results indicate that Gαi is a main mediator of the GnRHR–MAPK pathway in this system. However, this effect does not rule out the involvement of other components that may be involved specifically in the GnRH-induced JNK activation.

## 3. Discussion

**Figure 10:**
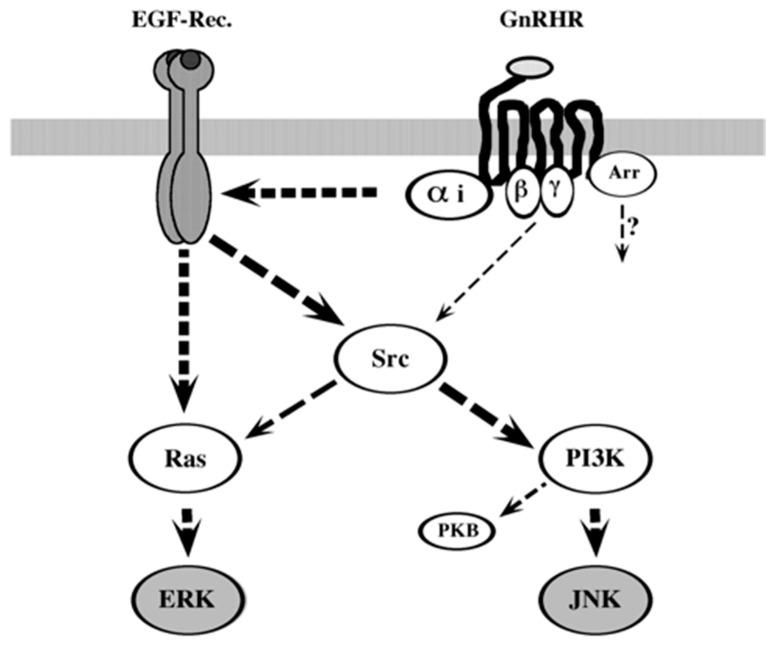
Schematic representation of GnRH signaling towards the MAPK cascades in transfected COS7 cells. Broken lines indicate indirect or weak activation and a solid lines indicate direct activation. Arr—β-arrestin.

GnRHR signaling is distinct among different cell lines and conditions, and therefore it may serve as a model for the variable GPCR signaling towards MAPKs [8]. Here, we delineated the GnRHR signaling when overexpressed in COS7 cells. These results are best explained by a model in which ERK GnRHR transmit its signal mainly via Gαi, which causes activation of the EGF receptor. At this stage, the pathways leading to the activation of the two MAPKs ERK and JNK diverge. ERK seems to be activated mainly by Ras, with some involvement of c-Src, but not of PI3K. On the other hand, JNK seems to be activated downstream of a pathway involving c-Src and PI3K (Figure 10). Interestingly, β-arrestin is involved in both pathways, likely operating via an independent mechanism. Other signaling moieties that were implicated in GPCR activity, including FAK, dynamin, and PKC, are not involved in the GnRH-MAPK signaling in GnRHR-expressing COS7 cells.

In previous studies, we elucidated the signaling mechanisms of GnRHR towards JNK and ERK in the pituitary-derived αT3-1 cells, in which the GnRHR is naturally expressed in its physiological role [29,30,32]. However, since the signaling may vary between distinct conditions, it was important to study other possible mechanisms that may transmit similar signals. For this purpose, we used GnRHR-transfected COS7 cells, in which GnRH does not have a natural role. Interestingly, the pathways elucidated in these cells were substantially different from those observed in other cells, shedding light on unique GPCR-induced signaling mechanisms. These findings are important mainly because they may clarify signaling pathways initiated by other GPCRs to activate the MAPK cascades, including the signaling by a putative second GnRHR that was previously described [52,53]. In addition, GnRH seems to have also extra-pituitary effects upon the gonads and gonadal steroid-dependent tumor cells [39], and our studies may help in dissecting signaling in these systems as well.

GnRHR signaling to the ERK cascades in αT3-1 cells has been extensively studied. This signaling was shown to involve a simultaneous activation of a Raf-1 by PKC, which is supported by a pathway that involves dynamin, c-Src, and Ras [30]. In addition, it was shown that elevation of Ca^2+^ levels, which may be derived from distinct sources, is involved in the activation of ERK by GnRH as well [29,34]. These distinct signaling mechanisms seem to form a signaling network and complement each other in their influence on MAPK activation. Therefore, it is likely that the distinct pathways identified in different laboratories are not contradictory, as they are all required to achieve the full GnRH effect on MAPKs. However, it is possible that the relative contribution of each of the pathways may change depending on the conditions. Parameters that may affect these pathways might be the changes in the frequency of GnRH pulses or in the varying conditions (e.g., stress) that may occur in the brain. Notably, in the αT3-1 cells, Gαq and PKC are the main signaling components that initiate the GnRHR–MAPK pathway [8]. However, these components do not participate in the signaling to MAPKs in the GnRHR-transfected COS7 cells. Rather, we found that the main signaling components in these cells are Gαi and EGF receptor, while β-arrestin and to some extent also the dissociated Gβγ dimer play complementary roles (Figure 10). It should be noted, however, that in different experimental systems, GnRHR may use distinct signaling pathways for MAPK activation. For example, in GGH3 cells, ERK activation is induced by PKA as well as by PKC [38]. In Caov-3 cells, GnRHR operates via Gαι/o, but also independently by Gβγ [39]. In LβT2 cells, JNK activation by GnRH is independent of PKC [54], and in immortalized GT1-7 neurons, GnRH transmits it signals through a pathway that includes EGF receptor activation by PKC [40,41]. Again, the results obtained here together with the results of other studies show that GnRH signaling may be mediated by distinct mechanisms in different cell types and under different conditions. However, despite this distinct signaling, the outcome of the signal is very often similar.

Interestingly, variability of GnRHR signaling was detected not only in distinct cell lines, but even within a particular cell line, probably under distinct conditions. In this study, we found the involvement of Gαi, EGF receptor, and to some extent also of c-Src and β-arrestin in the GnRHR–ERK pathway. However, Grosse et al. found that Gq and PKC are the main signaling components involved [33] in the transmission of the same signals in the same cells, while in αT3-1 cells the effect was mediated by the EGF receptor. These results are exactly opposite to our findings here and in αT3-1 cells [30]. Furthermore, GnRH-induced JNK activation in αT3-1 cells was shown by Mulvaney and Roberson [35] to involve elevation in intracellular Ca^2+^ levels, but not of PKC, which is distinct from our findings [32], showing that PKC is the main signal transducer of the GnRHR–JNK pathway in the same cells. We do believe that all of the results published here are correct for the particular αT3-1- and GnRHR-transfected COS7 cells. Therefore, it is likely that the signaling properties of similar cells are modified by distinct growing conditions. Indeed, we found that long-term maintenance of αT3-1 cells in culture may gradually modulate their signaling properties to become less dependent on PKC for ERK activation. In addition, inappropriate serum starvation results in irreproducible signaling results in both cell types, which might be mediated by unequal removal of MAPK phosphatases. Independent of the upstream pathways, the reason for the ability of the receptor to induce similar effects by distinct pathways is still not understood. However, it may be explained by the centrality of the MAPK cascades that receive inputs from distinct pathways to mediate similar outcomes. This assumption requires further studies.

We show here that both Gαi and EGF receptor participate in GnRHR signaling. Indeed, these two components seem to be important not only for GnRHR, but also for many other GPCRs [8]. However, the mechanism by which Gαi activates EGF receptor is not fully elucidated, and may vary in different systems. Among other mechanisms, this process was shown to be mediated by many distinct proteins, including Gαi-induced activation of c-Src, interaction of EGF receptor with the GPCR itself, interaction with Gβγ, and more [42]. Therefore, the mode of EGF receptor activation in our transfected COS7 cells seems to be unique, probably relying on a direct interaction with Gαi upon GnRHR stimulation.

One of the main components in the GnRHR–MAPK pathway is c-Src, which was shown to participate in the transduction of most other GPCRs signals [55]. This PTK was shown in different systems to act either solely or in cooperation with additional signaling components to activate the MAPK cascades [8,56,57,58]. As mentioned above for the EGF receptor, the mechanism by which c-Src is activated by GPCRs varies among distinct systems. Among others, it has been shown that c-Src is activated by a PKC-dependent mechanism [59], via interaction with dissociated Gβγ dimer [60], by an interaction with Gα protein [61], by interaction with the GPCRs themselves via the proline-rich motifs of the receptors [62], or by interaction with β-arrestin [63,64]. In many cases, the direct interactions occur through the SH3 domain of c-Src in order to open the folded kinase domain in a way to activate it. We have previously shown that in αT3-1 cells, c-Src is partially activated downstream of PKC [32] via a mechanism that may involve dynamin [30]. However, in the GnRHR-transfected COS7 cells, the PKC inhibitor GF109203X had no influence on c-Src activity. Rather, the activation of c-Src is dependent on activated EGF receptor (Figure 7), which might be supported by Gβγ, as in other systems [56]. To our knowledge, this is the first demonstration of a direct c-Src activation by RTKs downstream of GPCRs. Hence, c-Src activation by GPCRs can occur through transactivation of RTKs and is not solely involved in the RTK activation.

We identified here the involvement of PI3K activation in the GnRHR to JNK pathway. This lipid kinase has already been implicated in GPCR signaling [65], leading to the regulation of cell growth, survival, and malignant transformation [66]. More than one mechanism may be involved in PI3K activation downstream of GPCRs [8]. These include activation by dissociated Gβγ dimer [67,68], which is mediated by a direct interaction of the Gβγ dimer with the catalytic p110 subunit of PI3K [69]. In COS7 cells, it was shown that both Gαi and Gαq can induce PI3K activation [67], while gastrin receptor was shown to induce this activation via FAK [70] or PTK (Src-like)-induced phosphorylation of IRS-1, which leads to the recruitment and activation of PI3K [71]. Importantly, the signals may be mediated via EGF receptor [17], as PI3K can be activated by a direct interaction with this RTK, or alternatively with the Ras that is activated downstream of the RTK [72]. Here, we did not find evidence for any of the above processes in the activation of PI3K by GnRH. On the other hand, the inhibition of PKB downstream of PI3K by both Csk and the dominant-negative EGF receptor led to our conclusion that PI3K is activated in our system mainly via a pathway involving EGF receptor and c-Src. β-Arrestin is an additional signaling component that we found to participate to some extent in the activation of both JNK and ERK by GnRH in our system. Interestingly, this component is not involved in the activation of c-Src or PI3K, as these two enzymes were not influenced by the expression of dominant-negative EGF receptor. Therefore, it is likely that β-arrestin lies downstream of these two components in the activation of the two MAPKs. One possibility for this is that β-arrestin acts directly on the MAPK cascades, and indeed it was reported that this protein may interact directly with ERK and JNK [24,73]. Finally, other signaling components that have previously been shown to participate in GPCR signaling were not detected as signal transducers in our system. Among these proteins are Gαq, PKC, dynamin, and FAK, which are expressed in COS7 cells, but from some reason are not wired to the GnRHR-MAPK pathways. The mechanism that prevents their interaction requires further investigation.

In conclusion, we report here that in GnRHR-expressing COS7 cells, ERK and JNK are activated by two distinct pathways that are initiated by Gαi and the EGF receptor. Then, the EGF receptor activates c-Src and PI3K to lead to JNK activation, whereas ERK activation is mediated by Ras, either directly or via c-Src. In addition, the dissociated Gβγ dimer may support the activation of c-Src, while β-arrestin seems to operate via an independent mechanism. Importantly, these pathways are different from those detected in other cell lines. Therefore, we conclude that GPCR signaling may be influenced by the cellular contexts, independent of the exact signaling wiring, while the outcome at the level of MAPKs is still very similar. The reason for this independence of upstream wiring and the roles of the components identified here need further investigation.

## 4. Materials and Methods

### 4.1. Stimulants, Inhibitors, Antibodies and Miscellaneous Reagents

[D-Trp^6^]-GnRH, a stable GnRH analog (GnRH-a), genistein (PTK inhibitor), enolase, and protein A/G-sepharose were obtained from Sigma Chemical (St. Louis, MO, USA). GF109203X, PD098059, SB203580, wortmannin, AG1478, PP1, pertussis-toxin, anti-pan-ras monoclonal antibody, and TPA were purchased from Calbiochem. Polyclonal anti-ERK (C16, sc-093; 1:2500), anti-c-Src (sc-18; 1:1000), anti-phosphotyrosine (PY99, sc7020; 1:1000), anti-phospho EGFR (Tyr 1173, sc-101668; 1:2000), anti-EGFR (sc-03, 1:2000), and anti-Hb-EGF (C-18, sc-1413; 1:1000) antibodies were from Santa Cruz Biotechnology, Inc. Monoclonal anti-diphospho (DP)-ERK (M8159; 1:20,000) and DP-JNK (J4750; 1:2000) antibodies (anti-active ERK and JNK); polyclonal anti-phospho S473 protein kinase B (PKB) antibody (P4112, 1:2000); and polyclonal anti-general ERK (M5670, 1:5000), JNK (J4500; 1:1000), and PKB (P1601; 1:2000) antibodies were from Sigma, Israel (Rehovot, Israel). Polyclonal anti-phospho-(Tyr-416)-Src antibody (anti-active Src; #2101; 1:1000 dilution) was purchased from New England Biolabs.

### 4.2. Plasmids

GnRHR (mouse) was cloned into pCDNA1 using the BamHI/XbaI sites. Human FAK and N-terminally truncated FAK (Dn-FAK; FRNK; human) were cloned in pCDNA1 using the BamHI/XhoI sites. N-17 Ras in pCDNA1 and Csk-pRK5 were prepared as previously described [74]. Mammalian expression vectors containing wild-type dynamin, dominant-negative dynamin (K44A-dynamin), β-arrestin2 in pCMV5, and dominant-negative β-arrestin (V54D-β-arrestin2) in pCDNA3 were a gift from Dr. M. Caron, Duke University, NC. CD8-tagged βARK was from Dr. Zvi Vogel (Weizmann Institute of Science, Israel). Dn–EGF receptor (K721A) was provided by Dr. Y. Yarden (Weizmann Institute of Science).

### 4.3. Transfection, Stimulation, and Harvesting of COS7 Cell

Subconfluent COS7 cells were transfected with 5 µg of the GnRHR together with 5 µg of either an examined plasmid or vector control. The transfection was carried out using the DE-dextran technique [75], and the transfection efficiency was 80–95%. When time courses were determined, the transfected cells were pulled sixteen hours after transfection and split again on the assay plate to ensure homogeneous expression of GnRHR in all plates. Thirty-two hours after transfection, the cells were serum-starved for 16 h and incubated for the desired time intervals with GnRH-a in the presence or absence of various inhibitors. After stimulation, cells were washed twice with ice cold PBS and once with buffer A consisting of 50 mM β-glycerophosphate (pH 7.3), 1.5 mM EGTA, 1 mM EDTA, 1 mM DTT, 0.1 mM sodium orthovanadate. The cells were subsequently harvested in ice cold homogenization buffer (Buffer H) consisting of 50 mM β-glycerophosphate (pH 7.3), 1.5 mM EGTA, 1 mM EDTA, 1 mM DTT, 0.1 mM sodium orthovanadate, 1 mM benzamidine, aprotinin (10 µg/mL), leupeptin (10 µg/mL), and pepstatin (2 µg/mL). Cell lysates were centrifuged (20,000× *g*, 15 min) and the supernatant was assayed for protein content. For the determination of c-Src and EGF receptor, the cells were lysed with RIPA buffer consisting of 137 mM NaCl, 20 mM Tris, pH 7.4, 10% (*v*/*v*) glycerol, 1% Triton X-100, 0.5% (*w*/*v*) deoxycholate, 0.1% (*w*/*v*) SDS, 2.0 mM EDTA, 1.0 mM PMSF, and 20 µM leupeptin. After 10 min in the RIPA buffer (4 °C), the cell lysates were centrifuged (20,000× *g*, 15 min) and the supernatant was subjected to c-Src assay as below.

### 4.4. Western Blot Analysis

Cell supernatants, which contained cytosolic proteins, were collected, denatured by boiling in sample buffer for 5 min, and aliquots from each sample (20 µg) were separated on 10% SDS-PAGE followed by Western blotting with the appropriate antibodies. Alternatively, immunoprecipitated samples were boiled in sample buffer and subjected to SDS-PAGE and Western blotting. The blots were developed with alkaline phosphatase or horseradish peroxidase-conjugated anti-mouse or anti-rabbit Fab antibodies (Jackson).

### 4.5. Determination of ERK Activity

Transfected COS7 cells were serum-starved (0.1% FCS, 16 h) and the examined stimulants were added for various time intervals. The cells were then washed (twice with phosphate-buffered saline (PBS) and once with buffer A), scraped into 300 µL buffer H, and sonicated (50 W, 2 × 7 s) at 4 °C. After centrifugation (20,000× *g*, 15 min, 4 °C), aliquots of the resulting supernatant were subjected to immunoprecipitation with anti-ERK C terminal antibody using protein A or protein G agarose (20 µL). ERK activity was determined by the phosphorylation of myelin basic protein as described in [74]. 

### 4.6. Solid-Phase Assay for JNK Activity

Transfected COS7 cells were serum-starved (0.1% FCS, 16 h) and the examined stimulants were added for various time intervals. The cells were then washed (twice with phosphate-buffered saline (PBS) and once with buffer A), scraped into 250 µL of buffer H, and sonicated (50 W, 2 × 7 s), all at 4 °C. After centrifugation (20,000× *g*, 15 min, 4 °C), aliquots of the resulting supernatant were assayed by the Coomassie protein assay (Pierce) for protein. JNK activity was detected according to the method outlined by Hibi et al. [76]. Briefly, aliquots (100–150 µg protein) of the cell extracts were incubated (2 h, 4 °C) with GST-c-Jun (1–91) to allow the JNK to bind to the substrate. After extensive washing, the JNK activity was measured by phosphorylation of the GST-c-Jun (1–91), which was mediated by the bound kinase in the presence of 20 mM MgCl2, 20 µM [γ–^32^P]-ATP (300 cpm/pmol) for 20 min at 30 °C. The reactions were terminated by the addition of sample buffer and the samples were subjected to SDS-PAGE and autoradiography on Kodak X-100 films. The phosphorylation of GST-c-Jun (1–91) was quantitated by densitometry (Bio-Rad 690).

### 4.7. Determination of Ras Activity

Subconfluent COS7 cells were co-transfected with 5 µg of the GnRHR, together with 5 µg of either an examined plasmid or vector control. Thirty-two hours after transfection, the cells were serum-starved for 16 h and treated with GnRH-a (10^−7^M) for various time points in the absence or presence of the tested inhibitors (15 min, 37 °C). Following stimulation, the cells were lysed in Ral buffer (50 mM Tris-HCl, pH 7.5, 10% glycerol, 200 mM NaCl, 2.5 mM MgCl_2_, 1% NP-40, 1 µg/mL aprotinin, 1 µg/mL leupeptin, 0.1 mg/mL trypsin inhibitor, 250 µM PMSF, 10 mM NaF, and 1 mM sodium orthovanadate). After 10 min in the Ral buffer (4 °C), the cell lysates were centrifuged (14,000× *g*, 10 min) and the supernatant (300 µg protein) was subjected to further treatment. The active GTP-bound form of Ras was precipitated by GST-Raf-RBD (20 µg) and washed three times in a buffer containing 20 mM Hepes, pH 7.5, 0.15 M NaCl, 0.1% Triton X-100, and 10% glycerol, then washed once with buffer A. The amount of Ras pulled-down was assessed by Western blotting using mouse anti-pan-ras antibody.

### 4.8. Determination of PKB Activity

Transfected COS7 cells were serum-starved (0.1% FCS, 16 h) and the examined stimulants were added for various time intervals. The cells were then washed (twice with PBS and once with buffer A) and scraped into RIPA buffer. After centrifugation (20,000× *g*, 15 min, 4 °C), aliquots of the resulting supernatant were subjected to immunoprecipitation with anti-PKB C-terminal antibody using protein A agarose or protein G agarose (20 µL). PKB activity was determined by p-histone H2B as described for ERK above.

### 4.9. C-Src Activity

Cell lysates (400–500 µg protein in buffer H containing 1% Triton X-100) were incubated with anti-c-Src-antibody precoupled to protein-A Sepharose and swirled at 4 °C. The immunecomplexes were washed once with RIPA, twice with 0.5 M LiCl in 0.1 M Tris-HCl pH 8.0, and once with buffer A. The washed immunoprecipitates were resuspended in a kinase assay buffer [32] and the c-Src activity was determined using acid-denatured enolase (3 μM) as the substrate in the presence of 20 µM [γ^32^P]-ATP (8000 cpm/pmol). The enzymatic reactions were terminated by the addition of sample buffer. The samples were then subjected to SDS-PAGE and autoradiography. Alternatively, the harvested fractions were separated by SDS-PAGE and subjected to Western blot analysis with anti-active c-Src antibody and anti-general c-Src antibody.

### 4.10. Statistical Analysis

Data are expressed as the mean ± S.E.M of at least three repeats. Statistical evaluation was carried out using functional analysis and Student’s *t*-test (two-tailed) to test for differences between the control and experimental results. Values of *p* < 0.05 were considered statistically significant.

## Abbreviations

CAMKII	Calcium–calmodulin-dependent protein kinase II
DP	diphospho
Epac	cAMP-responsive Ras–guanine nucleotide exchange factor
ERK	extracellular signal-responsive kinase
FCS	fetal calf serum
GnRH	gonadotropin-releasing hormone
GnRHR	gonadotropin-releasing hormone receptor
GPCR	G-protein-coupled receptors
Hb	heparin binding
JNK	c-Jun N-terminal kinase
MAPK	mitogen-activated protein kinase
MBP	myelin basic protein
PBS	phosphate-buffered saline
PKB	protein kinase B
PKC	protein kinase C
PLC	phospholipase C
PTK	protein tyrosine kinase
RTK	receptor tyrosine kinase

## References

[1] Jain: R., Watson U., Vasudevan L., Saini D.K. (2018). ERK Activation Pathways Downstream of GPCRs. Int. Rev. Cell Mol. Biol..

[2] Preininger A.M., Meiler J., Hamm H.E. (2013). Conformational flexibility and structural dynamics in GPCR-mediated G protein activation: A perspective. J. Mol. Biol..

[3] Shimada I., Ueda T., Kofuku Y., Eddy M.T., Wuthrich K. (2019). GPCR drug discovery: Integrating solution NMR data with crystal and cryo-EM structures. Nat. Rev. Drug Discov..

[4] Kovacs J.J., Hara M.R., Davenport C.L., Kim J., Lefkowitz R.J. (2009). Arrestin development: Emerging roles for beta-arrestins in developmental signaling pathways. Dev. Cell.

[5] Keshet Y., Seger R. (2010). The MAP kinase signaling cascades: A system of hundreds of components regulates a diverse array of physiological functions. Methods Mol. Biol..

[6] Flores K., Yadav S.S., Katz A.A., Seger R. (2019). The Nuclear Translocation of Mitogen-Activated Protein Kinases: Molecular Mechanisms and Use as Novel Therapeutic Target. Neuroendocrinology.

[7] Lee S., Rauch J., Kolch W. (2020). Targeting MAPK Signaling in Cancer: Mechanisms of Drug Resistance and Sensitivity. Int. J. Mol. Sci..

[8] Naor Z., Benard O., Seger R. (2000). Activation of MAPK Cascades by G-protein-coupled Receptors: The Case of Gonadotropin-releasing Hormone Receptor. Trends Endocrinol. Metab..

[9] De Rooij J., Zwartkruis F.J., Verheijen M.H., Cool R.H., Nijman S.M., Wittinghofer A., Bos J.L. (1998). Epac is a Rap1 guanine-nucleotide-exchange factor directly activated by cyclic AMP. Nature.

[10] Campbell J.S., Seger R., Graves J.D., Graves L.M., Jensen A.M., Krebs E.G. (1995). The MAP kinase cascade. Recent Prog. Horm. Res..

[11] Nishizuka Y. (1992). Intracellular signaling by hydrolysis of phospholipids and activation of protein kinase C. Science.

[12] Kolch W., Heidecker G., Kochs G., Hummel R., Vahidi H., Mischak H., Finkenzeller G., Marme D., Rapp U.R. (1993). Protein kinase C alpha activates RAF-1 by direct phosphorylation. Nature.

[13] Farnsworth C.L., Freshney N.W., Rosen L.B., Ghosh A., Greenberg M.E., Feig L.A. (1995). Calcium activation of Ras mediated by neuronal exchange factor Ras-GRF. Nature.

[14] Sagasti A., Hisamoto N., Hyodo J., Tanaka-Hino M., Matsumoto K., Bargmann C.I. (2001). The camkii unc-43 activates the mapkkk nsy-1 to execute a lateral signaling decision required for asymmetric olfactory neuron fates. Cell.

[15] Jiang Y., Ma W., Wan Y., Kozasa T., Hattori S., Huang X.Y. (1998). The G protein G alpha12 stimulates Bruton’s tyrosine kinase and a rasGAP through a conserved PH/BM domain. Nature.

[16] Lopez I., Mak E.C., Ding J., Hamm H.E., Lomasney J.W. (2000). A novel bifunctional phospholipase C that is regulated by G{alpha}12 and stimulates the Ras/MAP kinase pathway. J. Biol. Chem..

[17] Daub H., Wallasch C., Lankenau A., Herrlich A., Ullrich A. (1997). Signal characteristics of G protein-transactivated EGF receptor. EMBO J..

[18] Mochizuki N., Ohba Y., Kiyokawa E., Kurata T., Murakami T., Ozaki T., Kitabatake A., Nagashima A., Matsuda M. (1999). Activation of the ERK/MAPK pathway by an isoform of rap1GAP associated with Galphai. Nature.

[19] Crespo P., Xu N., Simonds W.F., Gutkind J.S. (1994). Ras-dependent activation of MAP kinase pathway mediated by G-protein beta gamma subunits. Nature.

[20] Luttrell L.M., van Biesen T., Hawes B.E., Koch W.J., Touhara K., Lefkowitz R.J. (1995). G beta gamma subunits mediate mitogen-activated protein kinase activation by the tyrosine kinase insulin-like growth factor 1 receptor. J. Biol. Chem..

[21] Bell B., Xing H., Yan K., Gautam N., Muslin A.J. (1999). KSR-1 binds to G-protein betagamma subunits and inhibits beta gamma- induced mitogen-activated protein kinase activation. J. Biol. Chem..

[22] Daaka Y., Luttrell L.M., Ahn S., Della Rocca G.J., Ferguson S.S., Caron M.G., Lefkowitz R.J. (1998). Essential role for G protein-coupled receptor endocytosis in the activation of mitogen-activated protein kinase. J. Biol. Chem..

[23] Ahn S., Maudsley S., Luttrell L.M., Lefkowitz R.J., Daaka Y. (1999). Src-mediated tyrosine phosphorylation of dynamin is required for beta2-adrenergic receptor internalization and mitogen-activated protein kinase signaling. J. Biol. Chem..

[24] Luttrell L.M., Roudabush F.L., Choy E.W., Miller W.E., Field M.E., Pierce K.L., Lefkowitz R.J. (2001). Activation and targeting of extracellular signal-regulated kinases by beta -arrestin scaffolds. Proc. Natl. Acad. Sci. USA.

[25] Luttrell L.M., Hawes B.E., van-Biesen T., Luttrell D.K., Lansing T.J., Lefkowitz R.J. (1996). Role of c-Src tyrosine kinase in G protein-coupled Receptor and Gßg subunit-mediated activation of mitogen-activated protein kinase. J. Biol. Chem..

[26] Naor Z., Huhtaniemi I. (2013). Interactions of the GnRH receptor with heterotrimeric G proteins. Front. Neuroendocrinol..

[27] Bjelobaba I., Stojilkovic S.S., Naor Z. (2018). Editorial: Gonadotropin-Releasing Hormone Receptor Signaling and Functions. Front. Endocrinol..

[28] Naor Z., Shacham S., Harris D., Seger R., Reiss N. (1995). Signal transduction of the gonadotropin releasing hormone (GnRH) receptor: Cross-talk of calcium, protein kinase C (PKC), and arachidonic acid. Cell. Mol. Neurobiol..

[29] Reiss N., Levi L.N., Shacham S., Harris D., Seger R., Naor Z. (1997). Mechanism of mitogen-activated protein kinase activation by gonadotropin-releasing hormone in the pituitary of alphaT3-1 cell line: Differential roles of calcium and protein kinase C. Endocrinology.

[30] Benard O., Naor Z., Seger R. (2000). Role of dynamin, Src and Ras in the PKC-mediated activation of ERK by gonadotropin-releasing hormone. J. Biol Chem..

[31] Harris D., Chuderland D., Bonfil D., Kraus S., Seger R., Naor Z. (2003). Extracellular signal-regulated kinase and c-Src, but not Jun N-terminal kinase, are involved in basal and gonadotropin-releasing hormone-stimulated activity of the glycoprotein hormone alpha-subunit promoter. Endocrinology.

[32] Levi N.L., Hanoch T., Benard O., Rozenblat M., Harris D., Reiss N., Naor Z., Seger R. (1998). Stimulation of Jun N-terminal kinase (JNK) by gonadotropin-releasing hormone in pituitary alpha T3-1 cell line is mediated by protein kinase C, c-Src, and CDC42. Mol. Endocrinol..

[33] Grosse R., Roelle S., Herrlich A., Hohn J., Gudermann T. (2000). Epidermal growth factor receptor tyrosine kinase mediates Ras activation by gonadotropin-releasing hormone. J. Biol. Chem..

[34] Mulvaney J.M., Zhang T., Fewtrell C., Roberson M.S. (1999). Calcium influx through L-type channels is required for selective activation of extracellular signal-regulated kinase by gonadotropin-releasing hormone. J. Biol. Chem..

[35] Mulvaney J.M., Roberson M.S. (2000). Divergent signaling pathways requiring discrete calcium signals mediate concurrent activation of two mitogen-activated protein kinases by gonadotropin-releasing hormone. J. Biol. Chem..

[36] Vasilyev V.V., Lawson M.A., Dipaolo D., Webster N.J., Mellon P.L. (2002). Different signaling pathways control acute induction versus long-term repression of LHbeta transcription by GnRH. Endocrinology.

[37] Harris D., Bonfil D., Chuderland D., Kraus S., Seger R., Naor Z. (2002). Activation of MAPK cascades by GnRH: ERK and Jun N-terminal kinase are involved in basal and GnRH-stimulated activity of the glycoprotein hormone LHbeta-subunit promoter. Endocrinology.

[38] Han X.B., Conn P.M. (1999). The role of protein kinases A and C pathways in the regulation of mitogen-activated protein kinase activation in response to gonadotropin- releasing hormone receptor activation. Endocrinology.

[39] Kimura A., Ohmichi M., Kurachi H., Ikegami H., Hayakawa J., Tasaka K., Kanda Y., Nishio Y., Jikihara H., Matsuura N. (1999). Role of mitogen-activated protein kinase/extracellular signal-regulated kinase cascade in gonadotropin-releasing hormone-induced growth inhibition of a human ovarian cancer cell line. Cancer Res..

[40] Shah B.H., Soh J.W., Catt K.J. (2003). Dependence of gonadotropin-releasing hormone-induced neuronal MAPK signaling on epidermal growth factor receptor transactivation. J. Biol. Chem..

[41] Shah B.H., Farshori M.P., Jambusaria A., Catt K.J. (2003). Roles of Src and EGF receptor transactivation in transient and sustained ERK1/2 MAP kinase responses to GnRH receptor activation. J. Biol. Chem..

[42] Pierce K.L., Tohgo A., Ahn S., Field M.E., Luttrell L.M., Lefkowitz R.J. (2001). Epidermal growth factor (EGF) receptor-dependent ERK activation by G protein-coupled receptors: A co-culture system for identifying intermediates upstream and downstream of heparin-binding EGF shedding. J. Biol. Chem..

[43] Pierce K.L., Luttrell L.M., Lefkowitz R.J. (2001). New mechanisms in heptahelical receptor signaling to mitogen activated protein kinase cascades. Oncogene.

[44] Avidor-Reiss T., Nevo I., Levy R., Pfeuffer T., Vogel Z. (1996). Chronic opioid treatment induces adenylyl cyclase V superactivation. Involvement of Gbetagamma. J. Biol. Chem..

[45] Seger R., Krebs E.G. (1995). The MAPK signaling cascade. FASEB J..

[46] De Rooij J., Bos J.L. (1997). Minimal Ras-binding domain of Raf1 can be used as an activation-specific probe for Ras. Oncogene.

[47] Daub H., Weiss F.U., Wallasch C., Ullrich A. (1996). Role of transactivation of the EGF receptor in signalling by G-protein- coupled receptors. Nature.

[48] Maudsley S., Pierce K.L., Zamah A.M., Miller W.E., Ahn S., Daaka Y., Lefkowitz R.J., Luttrell L.M. (2000). The beta(2)-adrenergic receptor mediates extracellular signal-regulated kinase activation via assembly of a multi-receptor complex with the epidermal growth factor receptor. J. Biol. Chem..

[49] Prenzel N., Zwick E., Daub H., Leserer M., Abraham R., Wallasch C., Ullrich A. (1999). EGF receptor transactivation by G-protein-coupled receptors requires metalloproteinase cleavage of proHB-EGF. Nature.

[50] Stephens L.R., Eguinoa A., Erdjument-Bromage H., Lui M., Cooke F., Coadwell J., Smrcka A.S., Thelen M., Cadwallader K., Tempst P. (1997). The G beta gamma sensitivity of a PI3K is dependent upon a tightly associated adaptor, p101. Cell.

[51] Ho M.K., Wong Y.H. (2001). G(z) signaling: Emerging divergence from G(i) signaling. Oncogene.

[52] Troskie B., Illing N., Rumbak E., Sun Y.M., Hapgood J., Sealfon S., Conklin D., Millar R. (1998). Identification of three putative GnRH receptor subtypes in vertebrates. Gen. Comp. Endocrinol..

[53] Neill J.D., Duck L.W., Sellers J.C., Musgrove L.C. (2001). A gonadotropin-releasing hormone (gnrh) receptor specific for gnrh ii in primates. Biochem. Biophys. Res. Commun..

[54] Yokoi T., Ohmichi M., Tasaka K., Kimura A., Kanda Y., Hayakawa J., Tahara M., Hisamoto K., Kurachi H., Murata Y. (2000). Activation of the luteinizing hormone beta promoter by gonadotropin-releasing hormone requires c-Jun NH2-terminal protein kinase. J. Biol. Chem..

[55] Thomas S.M., Brugge J.S. (1997). Cellular functions regulated by Src family kinases. Annu. Rev. Cell Dev. Biol..

[56] Gutkind J.S. (1998). The pathways connecting G protein-coupled receptors to the nucleus through divergent mitogen-activated protein kinase cascades. J. Biol. Chem..

[57] Lefkowitz R.J. (1998). G protein-coupled receptors. III. New roles for receptor kinases and beta-arrestins in receptor signaling and desensitization. J. Biol. Chem..

[58] Luttrell L.M., Daaka Y., Lefkowitz R.J. (1999). Regulation of tyrosine kinase cascades by G-protein-coupled receptors. Curr. Opin. Cell Biol..

[59] Lu W.Y., Xiong Z.G., Lei S., Orser B.A., Dudek E., Browning M.D., MacDonald J.F. (1999). G-protein-coupled receptors act via protein kinase C and Src to regulate NMDA receptors. Nat. Neurosci..

[60] Luttrell L.M., Della Rocca G.J., van Biesen T., Luttrell D.K., Lefkowitz R.J. (1997). Gbetagamma subunits mediate Src-dependent phosphorylation of the epidermal growth factor receptor. A scaffold for G protein-coupled receptor-mediated Ras activation. J. Biol. Chem..

[61] Ma Y.C., Huang J., Ali S., Lowry W., Huang X.Y. (2000). Src tyrosine kinase is a novel direct effector of G proteins. Cell.

[62] Cao W., Luttrell L.M., Medvedev A.V., Pierce K.L., Daniel K.W., Dixon T.M., Lefkowitz R.J., Collins S. (2000). Direct binding of activated c-Src to the beta 3-adrenergic receptor is required for MAP kinase activation. J. Biol. Chem..

[63] Luttrell L.M., Ferguson S.S., Daaka Y., Miller W.E., Maudsley S., Della Rocca G.J., Lin F., Kawakatsu H., Owada K., Luttrell D.K. (1999). Beta-arrestin-dependent formation of beta2 adrenergic receptor-Src protein kinase complexes. Science.

[64] Miller W.E., Maudsley S., Ahn S., Khan K.D., Luttrell L.M., Lefkowitz R.J. (2000). beta-arrestin1 interacts with the catalytic domain of the tyrosine kinase c-SRC. Role of beta-arrestin1-dependent targeting of c-SRC in receptor endocytosis. J. Biol. Chem..

[65] Lopez-Ilasaca M., Crespo P., Pellici P.G., Gutkind J.S., Wetzker R. (1997). Linkage of G protein-coupled receptors to the MAPK signaling pathway through PI 3-kinase gamma. Science.

[66] Vanhaesebroeck B., Leevers S.J., Panayotou G., Waterfield M.D. (1997). Phosphoinositide 3-kinases: A conserved family of signal transducers. Trends Biochem. Sci..

[67] Murga C., Laguinge L., Wetzker R., Cuadrado A., Gutkind J.S. (1998). Activation of Akt/protein kinase B by G protein-coupled receptors. A role for alpha and beta gamma subunits of heterotrimeric G proteins acting through phosphatidylinositol-3-OH kinasegamma. J. Biol. Chem..

[68] Lopez-Ilasaca M., Gutkind J.S., Wetzker R. (1998). Phosphoinositide 3-kinase gamma is a mediator of Gbetagamma-dependent Jun kinase activation. J. Biol. Chem..

[69] Leopoldt D., Hanck T., Exner T., Maier U., Wetzker R., Nurnberg B. (1998). Gbetagamma stimulates phosphoinositide 3-kinase-gamma by direct interaction with two domains of the catalytic p110 subunit. J. Biol. Chem..

[70] Daulhac L., Kowalski-Chauvel A., Pradayrol L., Vaysse N., Seva C. (1999). Gastrin stimulates the formation of a p60Src/p125FAK complex upstream of the phosphatidylinositol 3-kinase signaling pathway. FEBS Lett..

[71] Daulhac L., Kowalski-Chauvel A., Pradayrol L., Vaysse N., Seva C. (1999). Src-family tyrosine kinases in activation of ERK-1 and p85/p110-phosphatidylinositol 3-kinase by G/CCKB receptors. J. Biol. Chem..

[72] Downward J. (1998). Mechanisms and consequences of activation of protein kinase B/Akt. Curr. Opin. Cell Biol..

[73] McDonald P.H., Chow C.W., Miller W.E., Laporte S.A., Field M.E., Lin F.T., Davis R.J., Lefkowitz R.J. (2000). Beta-arrestin 2: A receptor-regulated MAPK scaffold for the activation of JNK3. Science.

[74] Jaaro H., Rubinfeld H., Hanoch T., Seger R. (1997). Nuclear translocation of mitogen-activated protein kinase kinase (MEK1) in response to mitogenic stimulation. Proc. Natl. Acad. Sci. USA.

[75] Lopata M.A., Cleveland D.W., Sollner-Webb B. (1984). High level transient expression of a chloramphenicol acetyl transferase gene by DEAE-dextran mediated DNA transfection coupled with a dimethyl sulfoxide or glycerol shock treatment. Nucleic Acids Res..

[76] Hibi M., Lin A., Smeal T., Minden A., Karin M. (1993). Identification of an oncoprotein- and UV-responsive protein kinase that binds and potentiates the c-Jun activation domain. Genes Dev..

